# Gene Expression Profiling in Behcet's Disease Indicates an Autoimmune Component in the Pathogenesis of the Disease and Opens New Avenues for Targeted Therapy

**DOI:** 10.1155/2018/4246965

**Published:** 2018-04-24

**Authors:** Antonio Puccetti, Piera Filomena Fiore, Andrea Pelosi, Elisa Tinazzi, Giuseppe Patuzzo, Giuseppe Argentino, Francesca Moretta, Claudio Lunardi, Marzia Dolcino

**Affiliations:** ^1^Immunology Area, Pediatric Hospital Bambino Gesù, Viale San Paolo 15, 00146 Rome, Italy; ^2^Department of Experimental Medicine, Section of Histology, University of Genova, Via G.B. Marsano 10, 16132 Genova, Italy; ^3^Department of Medicine, University of Verona, Piazzale L.A. Scuro 10, 37134 Verona, Italy

## Abstract

Behçet disease (BD) is a chronic inflammatory multisystem disease characterized by oral and genital ulcers, uveitis, and skin lesions. Disease etiopathogenesis is still unclear. We aim to elucidate some aspects of BD pathogenesis and to identify specific gene signatures in peripheral blood cells (PBCs) of patients with active disease using novel gene expression and network analysis. 179 genes were modulated in 10 PBCs of BD patients when compared to 10 healthy donors. Among differentially expressed genes the top enriched gene function was immune response, characterized by upregulation of Th17-related genes and type I interferon- (IFN-) inducible genes. Th17 polarization was confirmed by FACS analysis. The transcriptome identified gene classes (vascular damage, blood coagulation, and inflammation) involved in the pathogenesis of the typical features of BD. Following network analysis, the resulting interactome showed 5 highly connected regions (clusters) enriched in T and B cell activation pathways and 2 clusters enriched in type I IFN, JAK/STAT, and TLR signaling pathways, all implicated in autoimmune diseases. We report here the first combined analysis of the transcriptome and interactome in PBCs of BD patients in the active stage of disease. This approach generates useful insights in disease pathogenesis and suggests an autoimmune component in the origin of BD.

## 1. Introduction

Behçet disease (BD) is a chronic multisystem disease mainly characterized by mucous-cutaneous lesions such as oral and genital ulcers, erythema nodosum-like lesions, and papulopustular lesions, and by uveitis. Moreover, manifestations of vascular, articular, neurologic, urogenital, gastrointestinal, pulmonary, and cardiac involvement may occur.

BD was first described by Hulusi Behçet in 1937 as a trisymptom complex represented by recurrent aphthous stomatitis, genital ulcers, and uveitis. The diagnosis of the disease is still based on clinical criteria since universally accepted pathognomonic laboratory tests are lacking. An international study group on Behcet's disease has recently revised the criteria for classification/diagnosis of BD [1].

There are sporadic cases of BD all around the world, but it is most frequently seen along the ancient Silk Route, with a prevalence of 14–20/100,000 inhabitants. According to epidemiological studies, the disease is most prevalent in countries located between 30 and 45° north latitude through the Mediterranean Basin, the Middle East and Far East regions such as China and Japan [2].

The interaction between a complex genetic background and both innate and adaptive immune systems leads to the clinical features of the disease. The presence of familiar cases in 10% of the patients, the particular geographic distribution and the high frequency of HLA-B51, a split antigen of HLA-B5, among a wide range of ethnic populations favours the role of genetic factors in the pathogenesis of the disease, but it remains poorly understood [3]. Non-HLA genes also contribute to the development of BD [3]. Genome-wide association studies have shown that polymorphisms in genes encoding for cytokines, activator factors, and chemokines are associated with increased BD susceptibility. Among cytokines, IL-10 polymorphisms cause a reduction of the serum level of IL-10, an inhibitory cytokine that regulates innate and adaptive immune responses; on the other hand, IL-23 receptor polymorphism, which reduces its ability to respond to IL-23 stimulation, is associated with protection from BD [3–5]. Recent data also reported associations with CCR1, STAT4, and KLRC4 encoding a chemokine receptor, a transcription factor implicated in IL-12 and IL-23 signaling and a natural killer receptor, respectively [6, 7]. Moreover, susceptibility genes implicating the innate immune response to microbial exposure have recently been identified by Immunochip analysis [8].

Increased Th1, CD4^+^, and CD8^+^ T cell, *γδ* ^+^ T cell, and neutrophil activities were found both in the serum and in inflamed tissues of BD patients, which suggests that innate and adaptive immunities are involved together in the pathogenesis of BD [2, 9]. Similar to other autoimmune disorders, BD shows Th1-type cytokine profiles. IL-2- and interferon- (INF-) γ-producing T cells were increased in patients with active BD, while IL-4-producing T cells were lower than in controls [10]. Recent findings have shown that Th17 may play an important role in the pathogenesis of the disease [2, 11]. This hypothesis is supported by the observation of high IL-21 and IL-17 levels in sera of patients affected by BD with neurologic involvement [12, 13]. Another study showed that Th17/Th1 ratio in peripheral blood of patients with BD was higher than those of healthy controls, whereas the Th1/Th2 and Th17/Th2 ratios were similar among the two groups. Patients with uveitis or folliculitis had higher Th17/Th1 ratio compared with patients without these manifestations [14, 15]. Further investigation is required in order to better understand the role of the immune system in BD and whether the polarization towards a Th1/Th17 pathway may play a critical role in BD pathogenesis.

In this study, we used a gene array strategy to identify transcriptional profiles of PBCs obtained from patients with active BD. Using this approach, we think we have been able to shed a new light on some aspects of the disease pathogenesis by dissecting different aspects of this complex pathology in order to better clarify the role of the immune system in BD.

## 2. Patients and Methods

### 2.1. Patients

We studied a cohort of 51 patients (16 males and 35 females, mean age: 37 ± 11 years) affected by BD, attending the Unit of Autoimmune Diseases at the University Hospital in Verona, Italy.

All patients fulfilled the International Criteria for Behçet Disease (ICBD): oral aphthosis, genital ulcers, and ocular lesions were each given 2 points, whereas 1 point was assigned to each of skin lesions, vascular manifestations, and neurological manifestations. A patient scoring 4 points or above was classified as having BD [16, 17].

At enrollment, none of the patients had active infections or was affected by malignancies.

A group of 10 subjects with BD was selected within the entire cohort of BD patients and utilized for the gene array study. The clinical features of the patients are reported in Table 1 that also includes a description of the BD patients selected for the gene array study.

A written informed consent was obtained from all the participants of the study. The study was approved by local Ethical Committee of the Azienda Ospedaliera Universitaria of Verona, Verona, Italy. All investigations have been conducted according to the principles expressed in the Helsinki declaration.

### 2.2. Gene Array

Blood sample collection was prepared using PAXgene Blood RNA tubes (PreAnalytiX, Hombrechtikon, Switzerland), and total RNA was extracted by following the manufacturer's instructions. cRNA preparation, sample hybridization, and scanning were performed as recommended by the Affymetrix (Affymetrix, Santa Clara, CA, USA) supplied protocols and by the Cogentech Affymetrix microarray unit (Campus IFOM IEO, Milan, Italy) using Human Genome U133A 2.0 (HG-U133A 2.0) GeneChip (Affymetrix). For gene expression profile analysis, we followed the methods of Dolcino et al. [18]. Trancripts with an expression level at least 2.0 fold different in the test sample versus control sample (*p* ≤ 0.01) were functionally classified according to the Gene Ontology (GO) annotations and submitted to the pathway analysis using the PANTHER expression analysis tools (http://pantherdb.org/) [19]. The enrichment of all pathways associated with the differentially expressed genes compared to the distribution of genes represented on the Affymetrix HG-U133A microarray was analyzed, and *p* values ≤ 0.05, calculated by the binomial statistical test, were considered as significant enrichment.

### 2.3. Protein-Protein Interaction (PPI) Network Construction and Network Clustering

The search tool for the retrieval of interacting genes (STRING version 1.0; http://string-db.org/) is an online database which includes experimental as well as predicted interaction information and comprises >1100 completely sequenced organisms [20]. DEGs were directly mapped to the STRING database for acquiring significant protein-protein interaction (PPI) pairs from a range of sources, including data from experimental studies and data retrieved by text mining and homology searches [21]. PPI pairs with the combined score of ≥0.7 were retained for the construction of the PPI network.

The graph-based Markov clustering algorithm (MCL) allows the visualization of high-flow regions (clusters/modules) separated by boundaries with no flow, containing gene products that are expected to be involved in the same (or similar) biological processes [22].

In order to detect highly connected subgraphs (areas), the MCL algorithm was applied to the protein interactome graph.

Cytoscape software [22] was used to visualize all the constructed networks.

### 2.4. PBMCs Isolation

PBMCs were obtained from 30 healthy donors and 30 patients affected by BD through a density-gradient centrifugation on Lymphoprep (Nycomed Pharma, Oslo, NO) at 800 ×g. Cells were washed twice with PBS and counted using acridine orange (Thermo Fisher Scientific, Waltham, MA, USA), considering only viable cells for FACS analyses.

### 2.5. FACS Analysis

Cell samples were treated by following the methods of Dolcino et al. [18]. Cells were stimulated over night with Dynabeads Human T-Activator CD3/CD28 (Life Technologies, Carlsbad, CA, USA). The detection of IL-17 production was analyzed using the IL-17 Secretion Assay (Miltenyi Biotec, Bergisch Gladbach), following the manufacturer's instruction as described in the methods of Dolcino et al. [18].

### 2.6. Real-Time RT-PCR

Total RNA was isolated from PBC using TRIzol Reagent (Invitrogen, Carlsbad, CA, USA), following the manufacturer's instructions. PCR was performed by following the methods of Dolcino et al. [18]. Predesigned, gene-specific primers and probe sets for each gene (CCL2, CXCL2, ICAM1, and IL-8) were obtained from Assay-on-Demand Gene Expression Products (Applied Biosystems).

### 2.7. Detection of Soluble Mediators in Sera of BD Patients and Healthy Controls

Serum levels of TNF alpha, IL-8, CXCL1, CCL2, CCL3, and CCL20 were detected using commercially available ELISA kits (Quantikine, R&D Systems, Minneapolis, MN, USA), according to the manufacturer's instructions in 51 BD patients when compared to the 30 normal healthy donors.

### 2.8. Statistical Analysis

Statistical testing was performed using SPSS Statistics 2 software (IBM, United States). Data obtained from the analysis of the soluble mediators and from the analysis of IL-17-positive CD4^+^ T cells in PBMCs were analyzed using the Student's unpaired *t*-test.

## 3. Results

### 3.1. Gene Array Analysis

In order to identify specific gene signatures typically associated with BD, we compared the gene expression profiles of 10 PBC samples obtained from 10 individual BD patients with 10 PBC samples obtained from healthy age- and sex-matched donors.

We found that 179 modulated genes complied with the Bonferroni-corrected *p* value criterion (*p* ≤ 0.01) and the fold change criterion (FC≥2), showing robust and statistically significant variation between healthy controls and BD PBC. In particular, 160 and 19 transcripts resulted to be up- and downregulated, respectively.

**Figure 1:**
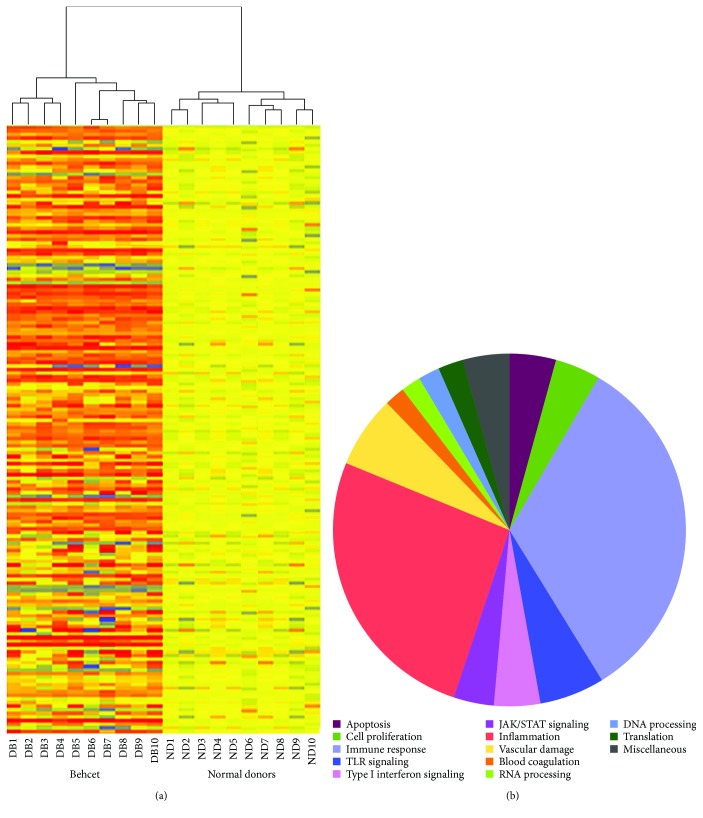
Modulated genes in PBCs of 10 BD patients and their functional classification. Heat map of significantly modulated genes (a). Each row represents a gene, each column shows the expression of selected genes in each individual sample. Blue-violet indicates genes that are expressed at lower levels when compared with the mean value of the control subjects, orange-red indicates genes that are expressed at higher levels when compared to the control means, and yellow indicates genes whose expression levels are similar to the control mean. Panel (b) shows the functional categorization of BD modulated genes according to GO terms. In the legend, the gene classes are listed in a clock-wise order starting at the “12 o'clock” position.

Figure 1(a) is a hierarchical cluster diagram representing the signal intensity of DEGs across samples; the heat map shows a different gene expression profile between BD patients and healthy donors that clearly separates the two sets of specimens.

Figure 1(b) shows a functional classification of all DEGs according to the Gene Ontology (GO) terms.

The Gene Ontology analysis showed that the vast majority of the regulated transcripts can be ascribed to biological processes that may play a role in BD, including inflammation, immune response, apoptosis, blood coagulation, vascular damage, and cell proliferation.  Table 2 shows a detailed selection of DEGs within the abovementioned processes. The table also includes GenBank accession numbers and fold changes. The complete list of modulated genes can be found in Supplementary Table 1.

Interestingly, regulated transcripts are distributed in gene categories that control different biological processes. However, the functional classes which show the highest enrichment in modulated genes are immune response (71/179) and inflammation (55/179).

Among genes ascribed to the immune response, twenty Th17-lymphocyte-related genes were upregulated including interleukin 6 signal transducer, IL6ST, chemokine (C-C motif) ligand 20, CCL20, suppressor of cytokine signaling 3, SOCS3, chemokine (C-X-C motif) ligand 1, CXCL1, chemokine (C-X-C motif) ligand 2, CXCL2, chemokine (C-X-C motif) ligand 3, CXCL3, inducible T cell costimulator, ICOS, intercellular adhesion molecule 1, ICAM1, interleukin 8, IL-8, interleukin 1 beta, and IL-1B (see also Table 2). Some genes involved in B cell activity (CD83 molecule, CD72 molecule, Fc receptor-like 2, FCRL2, and SAM domain, SH3 domain and nuclear localization signals 1 (SAMSN1)) are modulated in patients' samples, indicating a concomitant activation of this lymphocyte cell subset in BD.

Several upregulated genes play a role in innate immunity and are expressed in neutrophils (i.e., defensin, alpha 1, DEFA1, Fc fragment of IgA, receptor, and FCAR), dendritic cells (i.e., Dab, mitogen-responsive phosphoprotein, and homolog 2 DAB2), and in macrophages (adaptor-related protein complex 2, mu 1 subunit, and AP2M1).

In agreement with the typical presence of a marked inflammatory response in BD, we also observed overexpression of several proinflammatory transcripts. The upregulated genes comprise IL-8, IL-1B, CXCL2, CXCL1, CXCL3, interleukin 1, alpha (IL-1A), tumor necrosis factor (TNF), and oxidized low-density lipoprotein (lectin-like) receptor 1 (OLR1/LOX1).

Remarkably, in these two functional classes, we observed that a large number of genes are involved in well-known signaling networks that have been associated with autoimmune diseases.

These signal cascades include: (1) the interferon-alpha (IFN-A) pathway also named “type I interferon signature” [23], (2) the Toll-like receptor (TLR) signaling network, and (3) the JAK/STAT signaling pathway.

In particular, 9 type I interferon-inducible genes (IFIG) were upregulated (Table 2), thus showing the presence of an IFN type I signature, typically present in autoimmune diseases such as systemic lupus erythematosus (SLE), rheumatoid arthritis (RA), Crohn's disease, and Sjogren syndrome (SS) [24–30].

Twelve DEGs belong to the TLR signaling cascade (Table 2) which is thought to play a role in the onset of several autoimmune diseases and has been also implicated in the pathogenesis of BD [31–34].

Eight upregulated genes belong to the JAK/STAT signaling pathway, and interestingly, an increased JAK/STAT signaling has been associated with almost every autoimmune disease [35].

Moreover, activation of the JAK/STAT signaling pathway has been observed in monocytes and CD4^+^ T cells of patients with BD [36].

Several genes involved in apoptosis and/or in apoptosis regulation were modulated in BD samples including myeloid cell leukemia sequence 1 (BCL2-related), MCL-1, BCL2-like 11, BCL2L11, immediate early response 3, IER3 and ZFP36 ring finger protein-like 2, and ZFP36L2.

Cell proliferation was also deregulated, and we found modulation of several transcripts including BTG family, member 2, BTG2, epiregulin, EREG, proline-rich coiled-coil 2C (PRRC2), phosphatase, tensin homolog pseudogene 1 (PTENP1), and amyloid beta (A4) precursor-like protein 2 (APLP2).

Endothelial dysfunction and altered coagulation are typical features of BD vasculitis, and consistently with these aspects of the disease, several genes involved in vascular damage are modulated in BD specimens, including thrombospondin 1, THBS1; protein S alpha, PROS1; plasminogen activator, urokinase receptor, PLAU-UPAR; thrombomodulin, THBD; and vascular endothelial growth factor A, VEGFA.

**Figure 2:**
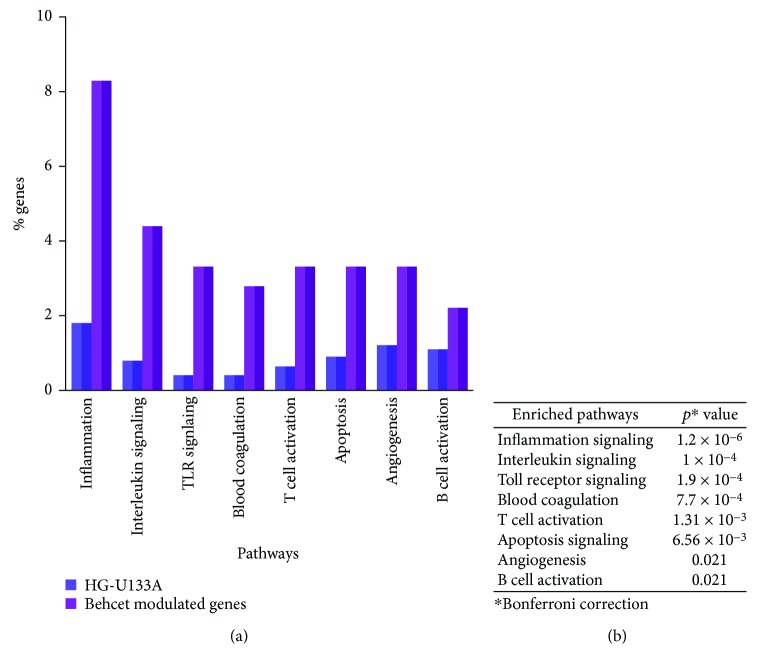
Pathways enrichment of BD modulated genes. (a) Graphical representation of genes ascribed to the enriched pathways, expressed in *y*-axis as percentage of all genes represented on the Affymetrix Human gene chip U133A 2.0 (dark purple bars) or as the percentage of genes in the dataset of Behcet's modulated genes (light purple bars); *x*-axis: enriched pathways. (b) *p* values associated with the significantly enriched pathways. Pathways with *p* values < 0.05 versus the distribution of all genes on the microarray chip, after a Bonferroni correction, were considered as significantly enriched.

The 179 DEGs were then submitted to a pathway analysis using the PANTHER expression analysis tool and functionally annotated according to canonical pathways. Eight canonical pathways were found to be significantly overrepresented among the differentially expressed genes, and inflammation was the most enriched pathways, followed by interleukin signaling, Toll-like receptor signaling, blood coagulation, T cell activation, apoptosis, angiogenesis, and the B cell activation pathway (Figure 2).

**Figure 3:**
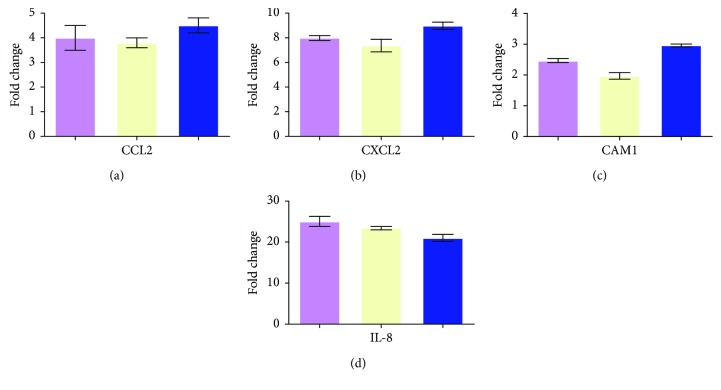
Real-time RT-PCR of some modulated genes confirms the results of gene array analysis. Genes selected for validation were CCL2, CXCL2, ICAM1, and IL-8. All the transcripts were increased in BD samples when compared to healthy donors. Relative expression levels were calculated for each sample after normalization against the housekeeping genes 18s rRNA, beta-actin, and GAPDH. Experiments have been conducted in triplicates. Housekeeping genes: violet bar: 18s rRNA; yellow bar: beta-actin; and blue bar: GAPDH.

The modulation of some genes showed by gene array analysis was validated by Q-PCR (Figure 3).

### 3.2. PPI Network Analysis

The gene expression profiling of BD PBC was then complemented by the study of functional interactions between DEGs' protein products.

To this aim, an interaction network was constructed upon the 179 DEGs, using the STRING data mining tool for retrieving well-documented connections between proteins. The obtained protein-protein interaction (PPI) network comprised 172 genes (nodes) and 2583 pairs of interactions (edges) (see Supplementary Figure 1).

When we performed a topological analysis of the PPI network using the Cytoscape software, we found that the number of interactions in which the products of the 71 “immune response genes” (see Supplementary Table 1) were involved, accounted for the vast majority (70%) of connections present in the whole network (1819/2583).

Given the high connectivity (i.e., number of connections) of the immune response gene products, we decided to perform an additional network analysis that focused on these gene products, thinking that they could be more informative.

**Figure 4:**
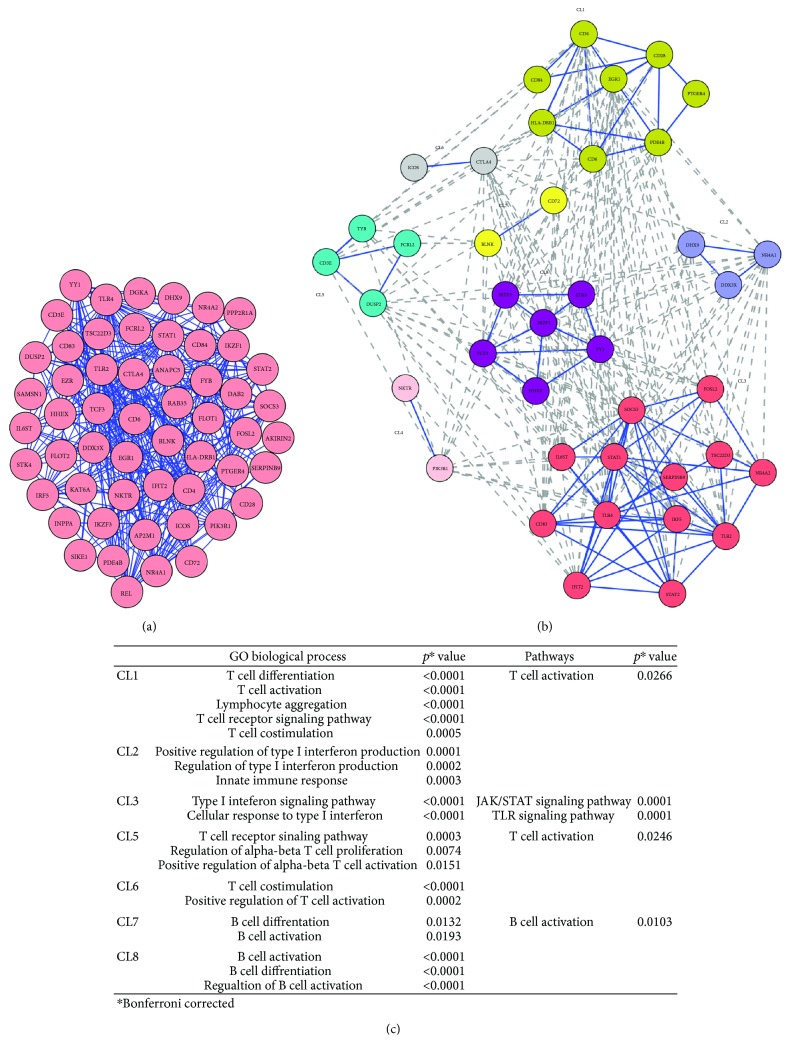
Network analysis of modulated genes in BD. Panel (a): PPI network of genes involved in immune response; panel (b): clusters extracted from the PPI network; panel (c): biological processes and pathways enriched in the eight modules.

We found that 55 proteins were linked into a complex network accounting for 307 pairs of interactions.  Figure 4(a) shows a graphical representation of the PPI network.

A clustering analysis was then carried out to detect clusters (modules) of proteins to which most of the interactions converged (“high flow areas”) using the MCL algorithm, and we identified eight clusters that collectively accounted for 40 nodes and 242 edges (Figure 4(b)).

We next performed a functional enrichment analysis to identify association of genes, in each cluster, with different “GO terms” and pathways.

The significantly enriched categories for each cluster are shown in Figure 4(c).

Interestingly, five out of eight clusters (CL1, CL5, CL6, CL7, and CL8) were representative of the adaptive immune response.

In particular, three clusters (CL1, CL5, and CL6) showed a statistically significant enrichment in “T cell-related” gene categories and included several genes typically associated with T cell-mediated immune responses such as: CD3E, CD4, CD6, CD28, CTLA4, and DUSP2.

The most enriched GO biological processes (GO-BP) in these clusters were: “T cell differentiation” and “T cell activation” (CL1), “T cell receptor signaling pathway” (CL5), “T cell costimulation”, and “positive regulation of T cell activation” (CL6). The most enriched pathway was the “T cell activation pathway” (CL1 and CL5).

Two clusters (CL7 and CL8) included DEGs typically associated with B cell functions (i.e., CD72). These clusters were significantly enriched in the GO-BP “B cell differentiation” (CL7) and “B cell activation” (CL7, CL8). Moreover, the B cell activation pathway was the top enriched pathway in cluster 7.

Several DEGs involved in the innate immune response (i.e., HDX9, DDX3X, SOCS3, STAT1, IRF5, IFIT2, and STAT2) were present in clusters CL2 and CL3. Interestingly, they were significantly enriched in functions of “positive regulation of type I interferon production” (CL2) and “type I interferon signaling pathway” (CL3), further confirming the presence of a type I interferon signature, typically associated with several autoimmune diseases. Moreover, genes in cluster 3 were significantly involved with the JAK/STAT signaling pathway (*p* = 0.0001) and the TLR signaling pathway (*p* = 0.0001), both implicated with the development of autoimmune diseases [31, 35]. Noteworthy, seven Th17-related proteins (CD28, CD4, ICOS, CD3E, YY1, TLR4, and IL6ST) were represented in the abovementioned clusters (CL1, CL3, CL5, CL6, and CL8). Finally, no significant GO-BP or pathway was identified in cluster CL4.

### 3.3. Frequency of IL-17-Positive CD4^+^ T Cells in PBMCs from Patients with BD

We assessed by flow cytometry the intracellular expression of the IL-17 cytokine, in PBMCs from 30 BD patients and from 30 healthy control subjects. We found a higher amount of IL-17-producing CD4^+^ T cells among the PBMCs of patients with BD compared with healthy controls.

**Figure 5:**
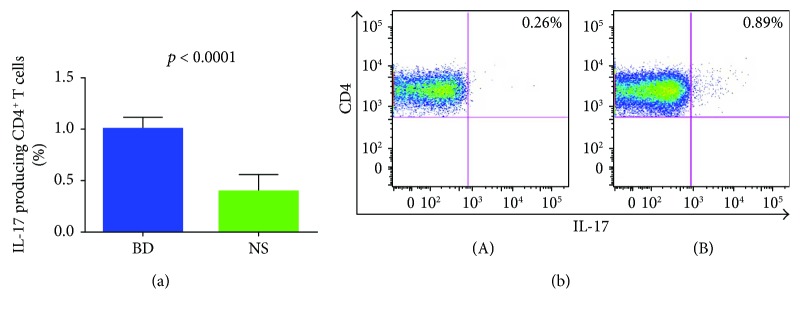
Flow cytometric analysis of CD4^+^ T cells releasing IL-17 in patients with BD. Panel (a) displays the mean percentage of CD4^+^ T cells releasing IL-17 of 30 healthy donors and 30 BD patients. PBMCs were stimulated over night with anti-CD3/CD28-coated beads. Panel (b) shows a representative experiment ((A) normal subjects (NS); (B) BD patients).

The mean values obtained in 30 BD PBMC were 1% ±0.12 versus 0.4% ±0.16 (*p* < 0.0001) (Figure 5).

### 3.4. Detection of Soluble Mediators in BD Sera

**Figure 6:**
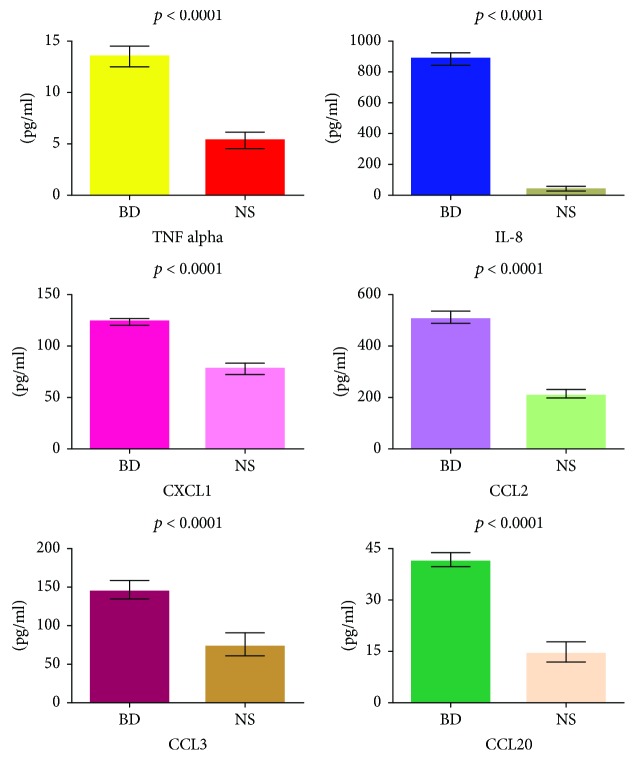
Serum levels of selected soluble mediators in BD patients and in normal subjects. The histograms represent the mean of the results obtained in 30 normal subjects (NS) and in 51 BD patients. *p* values were calculated using the Student's unpaired *t*-test.

The gene expression analysis was complemented by the detection of some of the corresponding soluble mediators in the sera of patients with BD. We chose to assess the levels of TNF alpha, IL-8, CXCL1, CCL2, CCL3, and CCL20.  Figure 6 shows the concentration of these molecules in the sera of the 51 BD patients. All these molecules showed increased serum levels in BD patients when compared to the 30 normal healthy donors.

## 4. Discussion

In this paper, we report a comprehensive study of BD gene expression profiling where for the first time, a conventional global gene expression analysis was combined to a gene network analysis of functional interactions between DEGs. We believe that this integrated approach is likely to generate insights in the complex molecular pathways that control the different clinical features of BD.

The first contribution of our study is a detailed investigation of DEGs in PBCs of BD patients in the attempt to clarify some aspects of BD pathogenesis.

Indeed, the majority of DEGs analyzed is involved in biological processes closely connected to the key features of the disease.

BD is a recurrent inflammatory disease with a multisystem involvement, affecting the vasculature, mucocutaneous tissues, eyes, joints, gastrointestinal tract, and brain.

Consistently with the strong inflammatory response typical of BD, DEGs indicate upregulation of a large number of proinflammatory molecules, including TNF, IL-1, IL-8, IL-10, CXCL1, CCL2, CCL3, and ICAM1, which can be detected at increased concentration in sera or plasma of BD patients when compared to healthy controls. [37–42].

Elevated serum levels of IL-8 are detectable in the active phase of BD and indicate the presence of vascular involvement [37], whereas high serum levels of CXCL1 correlate with BD disease activity [39]. Since flares of disease are characterized by neutrophil infiltration around blood vessels following increased chemotaxis of neutrophils [43], it is not surprising to observe upregulation of CSF3R/GCSFR, which controls neutrophil functions.

Consistently with the gene array data, serum levels of the proinflammatory mediators TNF alpha, IL-8, CXCL1, CCL2, CCL3, and CCL20 were significantly higher in our cohort of 51 BD patients when compared to healthy subjects.

The main histopathological finding in BD is a widespread vasculitis of blood vessels, arteries, and veins characterized by myointimal proliferation, fibrosis, and thrombus formation leading to tissue ischemia [44]. Occlusion of the vascular lumen creates a hypoxic milieu that effectively can induce new vessel formation. Angiogenesis can further stimulate inflammation since new born endothelial cells release chemoattractive mediators for leukocytes and express adhesion molecules [44]. Several DEGs play a role in angiogenesis, and the highest level of induction was observed for NOR1 (also named NR4A3), a gene expressed in developing neointima that promotes endothelial survival and proliferation, acting as a transcription factor in vascular development [45]. DEGs also showed upregulation of NAPA, known to induce VE-cadherin localization at endothelial junctions and regulate barrier function [46]. Interestingly, soluble VE-cadherins may be increased in the sera of BD patients [47]. Another upregulated transcript was the gene encoding for UPAR, expressed in several cell types including monocytes, neutrophils, activated T lymphocytes, macrophages, and endothelial cells. Indeed, high levels of the soluble form of UPAR have been detected in the plasma of BD patients [48].

Other genes, typically associated with the vasculitic process, were overexpressed in our array including THBD and PTX3. THBD can be detected at higher levels in sera of BD patients compared with healthy controls, and it is associated with the skin pathergy test, considered as a useful test for BD diagnosis. PTX3, an acute-phase reactant produced at sites of active vasculitis, is an indicator of active small vessel vasculitis [49].

Defects in blood coagulation and fibrinolysis have been described in patients with BD with or without thrombosis, and accordingly, we found downregulation of genes encoding for proteins that have an anticoagulant effect (i.e., TFPI, PROS1, and PROCR/EPCR) and upregulation of transcripts which promote the coagulation process (including THBS1, F5, and LMAN1).

Several DEGs indicate an altered apoptotic process with up- or downregulation of several apoptosis-related genes. Endothelial cell apoptosis which plays a pivotal role in vascular damage and autoantibodies which are able to induce endothelial cell apoptosis have been reported in BD [50]. In BD, an altered apoptosis has been described also in other cell subsets, that is, neutrophils and lymphocytes. Indeed, neutrophil apoptosis is reduced in the remission phase of uveitis and is restored in the active phase [51], whereas T lymphocytes are resistant to Fas-mediated apoptosis in BD with active uveitis [52]. On the contrary, an excessive expression of FasL on skin-infiltrating lymphocytes and the presence of apoptotic cells in the skin lesions have been also reported [53], suggesting that lymphocytes expressing increased levels of FasL may have a role in the development of BD skin lesions. Among genes that control cell proliferation, we observed overexpression of the gene EREG1, which plays an autocrine role in the proliferation of corneal epithelial cells [54], and APLP2 gene, involved in corneal epithelial wound healing [55]. In this regard, it is worthwhile mentioning that keratitis can be one of the ocular manifestations of BD.

Several aspects of BD are typical of an immune-mediated disease, but whether BD is an autoimmune or an autoinflammatory disease is still debated. A great number of DEGs (71/179) are involved in the immune response, and the majority of these genes can be ascribed to the adaptive immune response. In particular, DEGs indicate a T cell response with a prevailing upregulation of many TH17-related genes.

In this regard, it is worthwhile mentioning that Th17 cells have been associated with the pathogenesis of several autoimmune diseases including psoriasis, RA, and SLE [56–58]. Noteworthy, the involvement of this T cell subset in the pathogenesis of BD has been suggested, since Th17-related cytokines are considerably increased in BD and peripheral blood Th17/Th1 ratio is significantly higher in patients with active BD compared to healthy controls [11].

To further validate our data on overexpression of the Th17 pathway in our cohort of patients, we analyzed the presence of IL-17-producing CD4^+^ T cells and found a significantly increased percentage of these cells in PBCs of patients with BD when compared with healthy donors.

Among DEGs regulating B cell responses, we observed overexpression of SAMSN1, a transcript induced upon B cell activation [59], and EGR1, involved in the differentiation program of B cells into plasma cells, whereas the inhibitory receptor CD72 that downmodulates B cell receptor (BCR) signaling was downregulated. All together, these data indicate the activation of the B cell immune response and (auto)antibody production suggesting a possible role of these cells in BD pathogenesis.

Other genes associated with the adaptive immune response include ICOS, SOCS3, and HLA-DRB1. Interestingly, a high expression of ICOS on CD4^+^ T cells has been described in BD patients with active uveitis, suggesting a role in the pathogenesis of uveitis, possibly through upregulation of IFN-g, IL-17, and TNF [60].

An increased expression of SOCS3, a regulator of the JAK/STAT pathway of cytokine induction, has been observed in all patients with BD irrespective of disease activity [61], and polymorphisms of HLA-DRB1 alleles have been associated with BD [62].

In addition, it is worthwhile mentioning that DEGs of the adaptive immune response include transcripts already associated with the development of autoimmune diseases, including CTLA4, MST1, CD6, and the abovementioned SOCS3 [63–66].

We observed that several upregulated genes, including IRF5, IFIT2, DDX3X, STAT1, and STAT2 participate to type I interferon and JAK/STAT signaling pathways.

As already mentioned, type I interferon signaling is associated with autoimmune diseases including SLE, RA, Sjogren's syndrome, and Crohn's disease [24–30].

In this regard, the copresence of type I IFN signaling and Th17-related genes suggests an autoimmune component in the origin of BD, since a synergy between IFN and Th17 pathways is commonly involved in autoimmunity [24–30, 67–69].

Moreover, the JAK/STAT signaling is activated in BD [36] and this pathway has been associated with the development of systemic autoimmune diseases such as SLE and RA [70].

Our dataset indicates also the overexpression of several genes belonging to the TLR pathway. Growing body of evidence suggests the association between TLRs and autoimmunity. Indeed, the expression of TLRs in B cells is required for the synthesis of most of the SLE-associated autoantibodies [71]. Moreover, in RA, extracellular ligands can enhance the production of the proinflammatory mediators IL6 and IL-17 in human synoviocytes and in PBCs [72]. In addition, in systemic sclerosis activation of TLR4 on the surface of fibroblasts contributes to the upregulation of profibrotic chemokines [73]. Finally, stimulation of TLR2 induces the production of IL-23 and IL-17 cytokines from the PBCs of patients affected by Sjogren's syndrome [74].

TLR2 and TLR4 have been shown to be overexpressed in PBCs from patients with eye involvement [72] and in buccal mucosal biopsies and in PBCs obtained from patients with flare of the disease [32]. Noteworthy, both TLR2 and TLR4 were upregulated in our array, in accordance with the above reported data [32, 72].

Pathway analysis may help to elucidate the pathogenesis of complex or multifactorial diseases, such as BD, that are often caused by a mixture of abnormalities of correlated transcripts or biological pathways [75]. To this aim, we mapped our DEGs onto canonical pathways to identify signaling cascades which were overrepresented in our dataset.

Interestingly, pathway enrichment analysis revealed that inflammation, IL, TLR, blood coagulation, T cell activation, apoptosis, angiogenesis, and B cell activation signaling pathways were the most enriched in BD transcriptoma, further confirming their crucial role in the disease pathogenesis.

Thanks to our global analysis we have identified modulated genes involved in biological processes that could recapitulate most of the typical features of BD. Indeed, the majority of DEGs were involved in immune response and inflammation; moreover, we observed the activation of pathways (i.e., JAK/STAT and TLRs) and the presence of signatures (i.e., type I interferon and TH17 cell) typically associated with an autoimmune response, thus suggesting an autoimmune component in the origin of BD.

We carried our analysis in order to highlight key DEGs functionally collaborating in networks that could be involved in the disease onset and progression.

Indeed, in the second part of our study, instead of looking at single component of biological processes, we aim to study the interactions among the protein products of DEGs by a network analysis.

A network representation is an intriguing way to study the complex dynamic of disease-associated molecular interactions, and in this perspective, disorders can be considered in view of disturbances of molecular networks [76]. Interestingly, we observed that the protein products of genes ascribed to the immune response showed the highest degree of connectivity in the whole network of DEGs products, thus indicating a preeminent role of this gene category in driving the global gene expression profiles in BD pathogenesis. Then, we focused our attention on the PPI network specifically obtained from the immune response gene products, since it has been described that deregulation of genes, encoding for highly interactive proteins, interferes with physiological processes and that molecules involved in diseases development show a high attitude to interact with each other [77]. The clustering analysis of this sub-network helped us to further prioritize deregulated gene products that were placed in “highest connectivity areas” (clusters) of the network, where the hubs of biological process regulation are usually positioned [78].

In most of the clusters, we found an enrichment in molecular pathways of B and T cell-mediated adaptive immune response, thus suggesting a leading role of the adaptive immunity in the pathogenesis of BD. Moreover, DEGs in these classes included genes associated with the Th17 cell response.

Interestingly, we also observed that the molecules present in the few clusters enriched in innate immune response were involved in molecular signalings known to play a role in autoimmune diseases including JAK/STAT, TLRs, and type I interferon signaling.

These findings support that the disease may be sustained by an autoimmune process and are not in contrast with the hypothesis of an autoinflammatory component in the origin of BD.

The network analysis emphasizes the crucial role played by the molecular pathways emerged from our first global gene expression study in BD pathogenesis. Indeed, the molecules that participate to these signaling pathways are concentrated in areas (clusters) of the whole network that display the highest density of connection between genes, thus indicating their prominent role in the disease.

Through this analysis, we believe that we could identify pathogenically meaningful interactions that would have been hidden in the whole native dataset and that may be strongly associated with BD. Moreover, we provide evidence, at least at a level of gene expression, that BD may have an autoimmune origin.

Finally, we believe that our data can provide a deeper insight into BD pathogenesis, highlighting crucial molecular pathways including IL-17, IL-6, and JAK/STAT pathways that may be targeted by biological drugs and by novel therapeutical strategies.

## Figures and Tables

**Table 1:** Clinical features of the patients with BD included in the study.

Patients		51 (100%)

Sex	Male	16 (31%)
	Female	35 (68%)

Clinical features	Aphthous stomatitis	51 (100%)
	Genital ulcers	34 (66%)
	Erythema nodosum-like lesions	7 (13%)
	Papulopustular lesion	37 (72%)
	Uveitis	5 (9%)
	Epididymitis	3 (5%)
	Neurological symptoms	8 (14%)
	Vasculitis	6 (12%)
	Joints manifestations	43 (84%)
	Gastrointestinal involvement	3 (5%)
	Association with HLA-B51	32 (62%)

Patients utilised for gene array study		10 (100%)

Sex	Male	6
	Female	4

Clinical features	Aphthous stomatitis	10 (100%)
	Genital ulcers	4 (40%)
	Erythema nodosum-like lesions	1 (10%)
	Papulopustular lesion	8 (80%)
	Uveitis	1 (10%)
	Epididymitis	0
	Neurological symptoms	1 (10%)
	Vasculitis	3 (30%)
	Joint manifestation	8 (80%)
	Gastrointestinal involvement	0
	Association with HLA-B51	7 (70%)

**Table 2:** Annotated genes differentially expressed in BD PBC versus healthy controls grouped according to their function.

Probe set ID	Gene title	Gene symbol	FC	*p* value	Representative public ID
*Adaptive immune response*					
T cell response					
204794_at	Dual specificity phosphatase 2	DUSP2	2.12	0.001	NM_004418
216248_s_at	Nuclear receptor subfamily 4, group A, member 2	NR4A2	5.54	0.012	NM_006186
211861_x_at	CD28 molecule	**CD28**	2.00	0.014	AF222343
203547_at	CD4 molecule	**CD4**	2.02	0.016	BT019811
217394_at	T cell receptor alpha variable 13-1	TRAV13-1	2.53	0.001	AE000521
210439_at	Inducible T cell costimulator	**ICOS**	2.26	0.006	AB023135
221331_x_at	Cytotoxic T-lymphocyte-associated protein 4	CTLA4	2.13	0.003	NM_005214
211085_s_at	Serine/threonine kinase 4	STK4/MST1	2.14	0.005	Z25430
205456_at	CD3e molecule, epsilon (CD3-TCR complex)	**CD3E**	2.11	0.004	NM_000733
208602_x_at	CD6 molecule	CD6	2.47	<0.001	NM_006725
211302_s_at	Phosphodiesterase 4B, cAMP specific	PDE4B	3.17	0.004	L20966
218880_at	FOS-like antigen 2	FOSL2/FRA2	3.17	0.005	NM_005253
206360_s_at	Suppressor of cytokine signaling 3	**SOCS3**	2.01	0.001	NM_003955
212079_s_at	Lysine- (K-) specific methyltransferase 2A	KMT2A/MLL1	2.57	0.004	NM_001197104
209722_s_at	Serpin peptidase inhibitor, clade B (ovalbumin), member 9	SERPINB9	2.03	0.001	NM_004155
B cell response					
201694_s_at	Early growth response 1	EGR1	3.86	0.002	NM_001964
207655_s_at	B cell linker	BLNK	−2.10	<0.001	NM_013314
215925_s_at	CD72 molecule	CD72	−2.09	0.015	NM_001782
220330_s_at	SAM domain, SH3 domain, and nuclear localization signals 1	SAMSN1	2.03	0.006	NM_022136
204440_at	CD83 molecule	CD83	3.47		NM_004233
221239_s_at	Fc receptor-like 2	FCRL2	−2.36	0.001	NM_030764
213810_s_at	Akirin 2	AKIRIN2	3.12	<0.001	NM_018064
T/B cell response					
216901_s_at	IKAROS family zinc finger 1 (Ikaros)	IKZF1/IKAROS	6.08	<0.001	NM_006060
221092_at	IKAROS family zinc finger 3 (Aiolos)	IKZF3/AIOLOS	3.11	0.006	NM_012481
212249_at	Phosphoinositide-3-kinase, regulatory subunit 1 (alpha)	PIK3R1	3.19	<0.001	JX133164
*Innate immune response*					
201278_at	Dab, mitogen-responsive phosphoprotein, homolog 2	DAB2	−2.17	0.006	NM_032552
205033_s_at	Defensin, alpha 1	DEFA1	3.80	0.007	NM_004084
205468_s_at	Interferon regulatory factor 5	IRF5	2.01	<0.001	EF064718
M97935_5_at	Signal transducer and activator of transcription 1, 91 kDa	STAT1	2.26	0.001	GU211347
217199_s_at	Signal transducer and activator of transcription 2, 113 kDa	STAT2	2.02	0.002	S81491
217502_at	Interferon-induced protein with Tetratricopeptide repeats 2	IFIT2	2.00	<0.001	NM_001547
201211_s_at	DEAD (Asp-Glu-Ala-Asp) box polypeptide 3, X-linked	DDX3X	2.25	<0.001	NM_001356
211307_s_at	Fc fragment of IgA, receptor for	FCAR/CD89	5.04	0.004	U43677
212105_s_at	DEAH (Asp-Glu-Ala-His) box helicase 9	DHX9	3.82	<0.001	NM_001357
200613_at	Adaptor-related protein complex 2, mu 1 subunit	AP2M1	2.17	<0.001	NM_004068
NK cell response					
215339_at	Natural killer-tumor recognition sequence	NKTR	2.15	0.001	NM_005385
211242_x_at	Killer cell immunoglobulin-like receptor, two domains, long cytoplasmic tail, 4	KIR2DL4	2.00	0.004	AF276292
216552_x_at	Killer cell immunoglobulin-like receptor, two domains, short cytoplasmic tail, 4	KIR2DS4	2.08	0.002	NM_001281972
209722_s_at	Serpin peptidase inhibitor, clade B (ovalbumin), member 9	SERPINB9	2.03	0.001	NM_004155
*Adaptive/innate immune response*					
204863_s_at	Interleukin 6 signal transducer (gp130, oncostatin M receptor)	**IL6ST**	4.44	0.005	AB102799
211192_s_at	CD84 molecule	CD84	2.32	0.002	AF054818
213810_s_at	Akirin 2	AKIRIN2	3.12	0.001	AW007137
209722_s_at	Serpin peptidase inhibitor, clade B (ovalbumin), member 9	SERPINB9	2.03	0.001	NM_004155
221491_x_at	Major histocompatibility complex, class II, DR beta 1	HLA-DRB1	2.19	<0.001	U65585
213494_s_at	YY1 transcription factor	**YY1**	2.00	0.006	NM_003403
*Toll-like receptors signaling*					
205067_at	Interleukin 1, beta	IL-1B	6.32	0.001	NM_000576
206676_at	Carcinoembryonic antigen-related cell adhesion molecule 8	CEACAM8	5.07	0.003	M33326
204924_at	Toll-like receptor 2	TLR2	2.00	0.015	NM_003264
221060_s_at	Toll-like receptor 4	**TLR4**	2.10	0.001	NM_003266
211027_s_at	Inhibitor of kappa light polypeptide gene enhancer in B cells, kinase beta	IKBKB/IKKb	2.33	<0.001	AY663108
213281_at	Jun protooncogene	JUN/AP1	4.51	0.011	NM_002228
206035_at	V-rel reticuloendotheliosis viral oncogene homolog	REL/c-REL	2.56	0.005	NM_002908
217738_at	Nicotinamide phosphoribosyltransferase	NAMPT	2.64	0.008	NM_005746
216450_x_at	Heat shock protein 90 kDa beta (Grp94), member 1	HSP90B1/GP96	3.24	<0.001	NM_003299
214370_at	S100 calcium binding protein A8	S100A8	3.85	<0.001	NM_002964
211016_x_at	Heat shock 70 kDa protein 4	HSPA4	2.25	<0.001	NM_002154
211622_s_at	ADP-ribosylation factor 3	ARF3	2.12	<0.001	M33384
*Type I interferon signaling*					
205468_s_at	Interferon regulatory factor 5	IRF5	2.01	<0.001	EF064718
M97935_5_at	Signal transducer and activator of transcription 1, 91 kDa	STAT1	2.26	0.001	GU211347
217199_s_at	Signal transducer and activator of transcription 2, 113 kDa	STAT2	2.02	0.002	S81491
216598_s_at	Chemokine (C-C motif) ligand 2	CCL2	2.00	0.002	S69738
210001_s_at	Suppressor of cytokine signaling 1	SOCS1	2.16	0.012	AB005043
207433_at	Interleukin 10	IL-10	2.16	<0.001	NM_000572
210512_s_at	Vascular endothelial growth factor A	VEGFA	2.01	0.003	AF022375
217502_at	Interferon-induced protein with tetratricopeptide repeats 2	IFIT2	2.00	<0.001	NM_001547
201211_s_at	DEAD (Asp-Glu-Ala-Asp) box polypeptide 3, X-linked	DDX3X	2.25	<0.001	NM_001356
*JAK/STAT signaling*					
M97935_5_at	Signal transducer and activator of transcription 1, 91 kda	STAT1	2.26	0.001	GU211347
217199_s_at	Signal transducer and activator of transcription 2, 113 kda	STAT2	2.02	0.002	S81491
204863_s_at	Interleukin 6 signal transducer (gp130, oncostatin M receptor)	**IL6ST**	4.44	0.005	AB102799
207433_at	Interleukin 10	IL-10	2.16	<0.001	NM_000572
217489_s_at	Interleukin 6 receptor	**IL6R**	2.03	0.011	S72848
212249_at	Phosphoinositide-3-kinase, regulatory subunit 1 (alpha)	PIK3R1	3.19	<0.001	JX133164
210001_s_at	Suppressor of cytokine signaling 1	SOCS1	2.16	0.012	AB005043
206360_s_at	Suppressor of cytokine signaling 3	SOCS3	2.01	0.001	NM_003955
*Inflammatory response*					
207113_s_at	Tumor necrosis factor	**TNF**	2.00	0.008	NM_000594
211506_s_at	Interleukin 8	**IL-8**	10.44	0.013	AF043337
207433_at	Interleukin 10	**IL-10**	2.16	<0.001	NM_000572
205067_at	Interleukin 1, beta	**IL-1B**	6.32	0.001	NM_000576
217489_s_at	Interleukin 6 receptor	IL6R	2.03	0.011	S72848
209774_x_at	Chemokine (C-X-C motif) ligand 2	**CXCL2**	5.75	0.007	M57731
201939_at	Polo-like kinase 2	PLK2	6.20	0.012	NM_006622
204470_at	Chemokine (C-X-C motif) ligand 1	**CXCL1**	4.64	0.003	NM_001511
207850_at	Chemokine (C-X-C motif) ligand 3	**CXCL3**	3.53	0.015	NM_002090
210118_s_at	Interleukin 1, alpha	IL-1A	3.93	0.001	M15329
203751_x_at	Jun D protooncogene	JUND	3.39	0.008	NM_005354
216598_s_at	Chemokine (C-C motif) ligand 2	**CCL2**	2.00	0.002	S69738
205476_at	Chemokine (C-C motif) ligand 20	**CCL20**	2.23	0.011	NM_004591
205114_s_at	Chemokine (C-C motif) ligand 3	CCL3	2.21	0.007	NM_002983
210001_s_at	Suppressor of cytokine signaling 1	**SOCS1**	2.16	0.012	AB005043
212190_at	Serpin peptidase inhibitor, clade e, member 2	SERPINE2	−2.14	0.003	NM_006216
211919_s_at	Chemokine (C-X-C motif) receptor 4	CXCR4	2.06	0.008	AF348491
205099_s_at	Chemokine (C-C motif) receptor 1	CCR1	2.14	0.002	NM_001295
207075_at	NLR family, pyrin domain containing 3	NLRP3	2.15	0.011	NM_004895
203591_s_at	Colony-stimulating factor 3 receptor	CSF3R	1.89	0.012	NM_000760
215485_s_at	Intercellular adhesion molecule 1	**ICAM1**	2.04	0.015	NM_000201
209701_at	Endoplasmic reticulum aminopeptidase 1	ERAP1	2.44	<0.001	NM_016442
216243_s_at	Interleukin 1 receptor antagonist	IL-1RN	2.26	0.008	NM_173842
202643_s_at	Tumor necrosis factor, alpha-induced protein 3	TNFAIP3	2.15	0.012	NM_001270508
201044_x_at	Dual specificity phosphatase 1	DUSP1	4.71	0.013	NM_004417
210004_at	Oxidized low-density lipoprotein (lectin-like) receptor 1	OLR1/LOX1	4.13	0.002	AF035776
214370_at	S100 calcium binding protein A8	S100A8	3.85	<0.001	AW238654
213281_at	Jun protooncogene	JUN/AP1	4.51	0.011	NM_002228
216450_x_at	Heat shock protein 90 kDa beta (Grp94), member 1	HSP90B1/GP96	3.24	<0.001	NM_003299
*Vascular damage*					
Blood coagulation					
204614_at	Serpin peptidase inhibitor, clade B (ovalbumin), member 2	SERPINB2	3.96	0.013	NM_002575
202833_s_at	Serpin peptidase inhibitor, clade A, member 1	SERPINA1	2.25	<0.001	NM_000295
207808_s_at	Protein S alpha	PROS1	−2.16	0.002	NM_000313
201110_s_at	Thrombospondin 1	THBS1	5.08	0.001	NM_003246
204713_s_at	Coagulation factor V (proaccelerin, labile factor)	F5	2.53	0.001	NM_000130
203294_s_at	Lectin, mannose binding, 1	LMAN1	2.50	<0.001	U09716
213258_at	Tissue factor pathway inhibitor (lipoprotein-associated coagulation inhibitor)	TFPI	−2.53	0.001	BF511231
203650_at	Protein C receptor, endothelial	PROCR	−2.67	0.001	NM_006404
Angiogenesis					
211924_s_at	Plasminogen activator, urokinase receptor	PLAUR/UPAR	3.42	0.007	NM_002659
207329_at	Matrix metallopeptidase 8 (neutrophil collagenase)	MMP8	2.42	0.012	NM_002424
210512_s_at	Vascular endothelial growth factor A	VEGFA	2.01	0.003	AF022375
209959_at	Nuclear receptor subfamily 4, group A, member 3	NR4A3/NOR1	5.69	<0.001	U12767
208751_at	N-Ethylmaleimide-sensitive factor attachment protein, alpha	NAPA	2.56	0.001	XM_011527436
Vasculitis					
203887_s_at	Thrombomodulin	THBD	2.00	0.015	NM_000361
206157_at	Pentraxin 3, long	PTX3	2.22	0.001	NM_002852
218880_at	FOS-like antigen 2	FOSL2/FRA2	3.17	0.005	NM_005253
*Apoptosis*					
M97935_5_at	Signal transducer and activator of transcription 1, 91 kDa	STAT1	2.26	0.001	GU211347
200796_s_at	Myeloid cell leukemia sequence 1 (BCL2-related)	MCL1	8.02	<0.001	AF118124
200664_s_at	DnaJ (Hsp40) homolog, subfamily B, member 1	DNAJB1/HSP40	2.20	0.010	NM_006145
208536_s_at	BCL2-like 11 (apoptosis facilitator)	BCL2L11	2.38	0.001	NM_006538
213606_s_at	Rho GDP dissociation inhibitor (GDI) alpha	ARHGDIA	2.30	<0.001	NM_001185077
219228_at	Zinc finger protein 331	ZNF331/RITA	2.50	0.004	NM_018555
209722_s_at	Serpin peptidase inhibitor, clade b (ovalbumin), member 9	SERPINB9	2.03	0.001	NM_004155
201631_s_at	Immediate early response 3	IER3	2.85	0.001	NM_003897
201367_s_at	ZFP36 ring finger protein-like 2	ZFP36L2	9.07	<0.001	NM_006887
*Cell proliferation*					
201235_s_at	BTG family, member 2	BTG2	4.53	<0.001	U72649
205767_at	Epiregulin	EREG	3.51	0.013	NM_001432
214052_x_at	Proline-rich coiled-coil 2C	PRRC2C/XTP2	3.11	<0.001	NM_015172
208701_at	Amyloid beta (A4) precursor-like protein 2	APLP2	2.64	<0.001	NM_001642
217494_s_at	Phosphatase and tensin homolog pseudogene 1	PTENP1	3.11	0.001	AF040103

Bold characters indicate TH17-related genes.

## Supplementary Materials

Supplementary 1 Supplementary Table 1: annotated genes differentially expressed in BD PBCs versus healthy controls grouped according to their function.

Supplementary 2 Supplementary Figure 1: PPI network of modulated genes in BD PBCs.
