# Supplementary figures and images for: Gene Expression Profiling in Behcet's Disease Indicates an Autoimmune Component in the Pathogenesis of the Disease and Opens New Avenues for Targeted Therapy
Source: J Immunol Res. 2018 Apr 24;2018:4246965. doi: 10.1155/2018/4246965 (PMC5941805; doi:10.1155/2018/4246965)

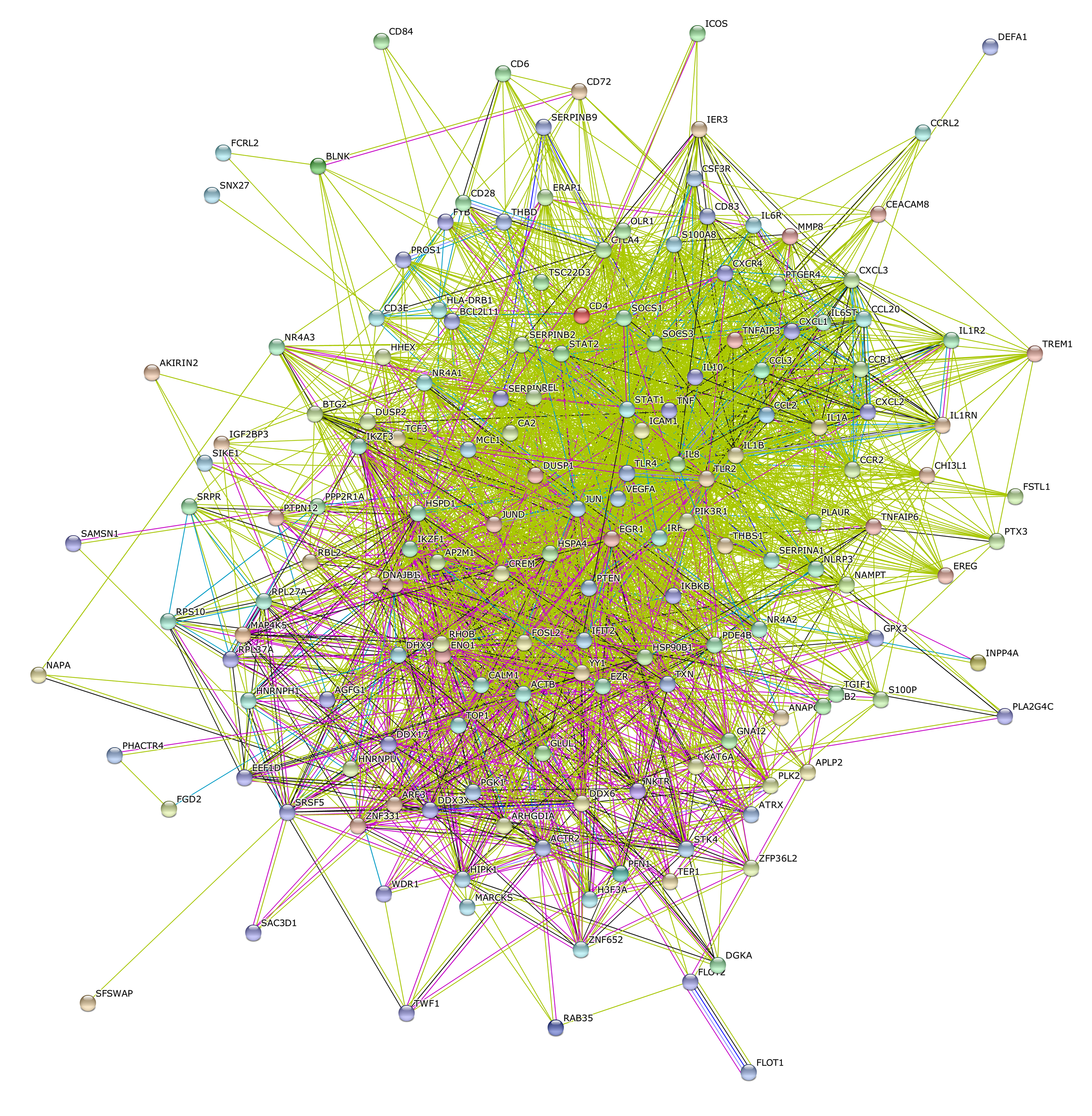

Supplement: Supplementary 1 — Supplementary Table 1: annotated genes differentially expressed in BD PBCs versus healthy controls grouped according to their function. [file 4246965.f1.tif]
